# RWDisEnh+: Enhancing disease-enhancer association prediction through multiplex-heterogeneous networks

**DOI:** 10.1371/journal.pone.0341861

**Published:** 2026-02-20

**Authors:** Duc-Hau Le

**Affiliations:** School of Information and Communications Technology, Hanoi University of Science and Technology, Hanoi, Vietnam; University of Illinois at Urbana-Champaign, UNITED STATES OF AMERICA

## Abstract

Enhancers are critical regulatory DNA elements that, when dysregulated, can contribute to disease pathogenesis by altering gene expression. Although millions of enhancers have been identified through large-scale genomic projects, their associations with human diseases remain largely uncharacterized, emphasizing the need for robust computational approaches. In our previous work, we developed RWDisEnh, a network-based method that integrates a shared gene–based enhancer network with a disease similarity network within a heterogeneous framework to predict disease–enhancer associations. In this study, we present RWDisEnh+ , an enhanced version of RWDisEnh that incorporates a sequence-based enhancer similarity network into a multiplex-heterogeneous network to improve prediction performance. Using an extended random walk with restart (RWR) algorithm, RWDisEnh+ allows information to propagate across disease and enhancer layers, leveraging both gene-based and sequence-based similarity features to rank candidate enhancers for each disease. Comprehensive evaluation using 3-fold cross-validation demonstrated that RWDisEnh+ achieves an average AUC of 0.874, outperforming RWDisEnh’s AUC of 0.819. Moreover, RWDisEnh+ identifies a larger number of evidence-supported disease–enhancer associations across top-k rankings, including 10 enhancers linked to seven diseases such as asthma, rheumatoid arthritis, and type 2 diabetes. GWAS validation and pathway enrichment analyses further reveal that these predicted associations are enriched in immune, inflammatory, and metabolic pathways, highlighting their biological relevance. Overall, RWDisEnh+ provides a stable and effective framework for predicting novel disease–enhancer associations, offering new insights into enhancer-mediated gene regulation and the genetic architecture of complex diseases.

## 1. Introduction

Enhancers are DNA sequences that regulate gene expression by binding to transcription factors, thereby increasing the transcription of a related gene. Genetic alterations in enhancers play a key role in the onset of complex diseases, as they have been demonstrated to promote disease advancement [1]. Recent reviews have comprehensively summarized the roles of enhancer dysregulation in disease and potential therapeutic strategies targeting enhancer function [2]. For instance, a mutation A > G at enhancer chr12:66581616–66581616 causes an increase in the expression level of gene IRAK3, subsequently leading to the development of acute lung injury [3]. An amplification (i.e., a copy number variant) in enhancers of gene KLF5 (chromosome 13) causes esophageal carcinoma [4]. A short indel at enhancer chr1: 46476824−46476824, which regulates gene MAST2, contributes to the development of breast cancer [5].

Large-scale efforts by consortia like ENCODE [6], FANTOM [7,8], and the NIH Epigenome Roadmap [9] have cataloged over three million enhancers using advanced computational techniques [10]. Beyond their roles documented in databases such as EnhancerAtlas [11], GeneHancer [12], and McEnhancer [13], the disease relevance of enhancers is increasingly recognized [14]. Yet, research linking enhancers to diseases has predominantly examined them individually [3–5]. Some disease-enhancer databases such as DiseaseEnhancer [15], EnDisease [16], ENdb [17], and CancerEnD [18] have begun consolidating these associations from existing literature, though only a limited subset of enhancers is currently tied to diseases. This gap highlights the urgent need for computational methods to systematically predict novel disease–enhancer associations.

In our earlier work [19], we proposed RWDisEnh, a random walk–based framework for predicting disease-associated enhancers, under the assumption that enhancers regulating common target genes are more likely to be involved in biologically related diseases [20]. The method constructed a heterogeneous network (HetNet) integrating enhancer–enhancer relationships (derived from shared target genes), disease–disease similarities, and known disease–enhancer associations. By applying a random walk with restart (RWR) on this integrated structure, RWDisEnh prioritized candidate disease–enhancer pairs and demonstrated superior performance compared with network diffusion (PageRank with Priors) and neighborhood-based (MaxLink) approaches [21–23].

In this study, we present RWDisEnh+ , an improved version of RWDisEnh that incorporates additional enhancer relationship information through a sequence-based enhancer similarity network (sEnhNet). In this network, similarities between enhancer sequences are computed using Clustal Omega [24] after converting genomic coordinates to FASTA sequences via BEDtools [25]. Unlike the shared gene-based enhancer network (gEnhNet), which captures functional associations through common target genes, sEnhNet leverages genomic sequence similarity to identify enhancers with comparable regulatory potential—thus revealing relationships overlooked by gene-centric approaches.

To comprehensively model enhancer–disease interactions, we integrate gEnhNet and sEnhNet into a multiplex network of enhancers (MulNet) and further connect it with the disease similarity network to form a multiplex-heterogeneous network (MulHetNet). The RWR algorithm is extended to operate on this multiplex-heterogeneous framework, introducing three key parameters: the restart probability (γ), the inter-layer jumping probability between enhancer networks (δ), and the importance weight of each enhancer network (τ).

Experimental results demonstrate that RWDisEnh+ achieves stable and superior predictive performance across parameter settings, with an average AUC of 0.874 on MulHetNet compared with 0.819 on HetNet (RWDisEnh) under 3-fold cross-validation. Moreover, RWDisEnh+ identifies more evidence-supported disease–enhancer associations, particularly for immune and metabolic diseases such as rheumatoid arthritis and type 2 diabetes. Subsequent GWAS and pathway enrichment analyses confirm the biological relevance of these predictions, linking novel enhancer–disease pairs to immune, inflammatory, and metabolic pathways. Collectively, RWDisEnh+ provides a more comprehensive and robust framework for predicting novel disease-associated enhancers, offering deeper insights into enhancer-mediated regulatory mechanisms and the genetic architecture of complex diseases.

## 2. Materials and methods

**Fig 1:**
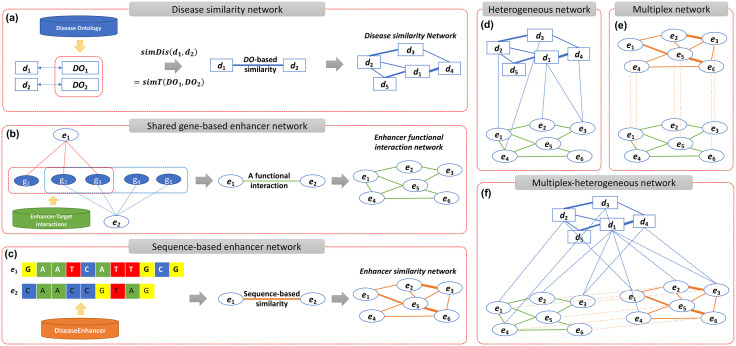
Development of disease and enhancer networks. **(a)** A disease similarity network was created using similarities between all pairs of Disease Ontology (DO) terms. **(b)** A shared-gene enhancer network was established by linking enhancer pairs with notably overlapping target genes. **(c)** A sequence-based enhancer similarity network was formed by connecting enhancer pairs based on their sequence similarities. **(d)** A heterogeneous network was constructed by integrating the shared-gene enhancer network, the disease similarity network, and known disease-enhancer associations. **(e)** A multiplex network is composed of the two enhancer networks. **(f)** A multiplex-heterogeneous network was formed by connecting the disease similarity network and the multiplex network by known disease-enhancer associations.

In this section, we first describe the databases that were used to construct all networks of diseases and enhancers and for all experiments. Then, we show how to build networks of diseases and enhancers (Fig 1). Finally, we introduce an improvement of our previous method, namely *RWDisEnh+*, for the prediction of disease-associated enhancers.

### 2.1 Databases

The Disease Ontology (DO) serves as a standardized vocabulary database, offering consistent, reusable, and sustainable definitions of human disease terms and related medical concepts for the biomedical community [26]. For the DO-based disease similarity network, we computed similarities between pairs of the 2,161 DO terms annotated in the DGA database [27] (Section 2.2.1 A disease similarity network).

We further gathered 1,059 established disease–enhancer associations from the DiseaseEnhancer database [15], a curated repository of manually verified links between 784 enhancers and 167 human diseases. Disease names were aligned with DO terms prior to calculating pairwise disease similarities; thus, we derived 959 associations between 122 DO terms and 738 enhancers. Ultimately, 58 DO terms representing diseases available in the DO-based disease similarity network were valid for analysis.

Additionally, the DiseaseEnhancer database provides enhancer information, including their chromosomal positions (start and end coordinates) and associated target genes. These enhancer–gene relationships were used to construct the gEnhNet, which implicitly reflects chromatin-level regulatory interactions. Chromosomal coordinates of enhancers were converted to genomic sequences for constructing the sEnhNet. Although other databases such as EnDisease [16], ENdb [17], and CancerEnD [18] exist, they have not been updated since their initial release and primarily focus on cancer-related enhancers. Therefore, DiseaseEnhancer remains the most suitable and comprehensive resource for constructing both enhancer–gene and disease–enhancer networks.

### 2.2 Construction of networks of diseases and enhancers

#### 2.2.1 A disease similarity network.

To construct the DO-based disease similarity network, we first mapped all diseases in the DGA database to their corresponding DO terms, resulting in 2,152 annotated terms among 2,161 DO terms. Disease Ontology (DO) organizes diseases as a directed acyclic graph (DAG), in which each term represents a disease concept, and parent–child relationships capture biological or clinical hierarchy.

The similarity between two DO terms (*t_i_* and *t_j_*) was computed using the Resnik semantic similarity approach [28], which quantifies how closely two ontology terms are related based on their most informative common ancestor (MICA) in the DO hierarchy. The information content (IC) of a term *t* is defined as:


IC(t)= −log(p(t))

where *p(t)* is the probability of term *t* occurring in the DGA database, calculated as: $p(t)=\frac{f(t)}{f(root)}$ with *f(t)* denoting the number of gene annotations under *t* (including its descendants) and *root* representing the top-level DO term (“disease”). The similarity between two DO terms is then defined as:


$$sim\left(t_{i},t_{j}\right)=\underset{c\in P\left(t_{i},{\;t}_{j}\right)}{max}\left(IC(c)\right)$$

where *P(t*_*i*_*, t*_*j*_*)* is the set of shared ancestors of *t*_*i*_ and *t*_*j*_.

For two diseases *d*_*i*_ and *d*_*j*_ directly mapped to *t*_*i*_ and *t*_*j*_, their semantic similarity is expressed as:


$$w_{ij}=simDis\left(d_{i},d_{j}\right)=sim\left(t_{i},t_{j}\right)$$

All disease pairs with $simDis\left(d_{i},d_{j}\right)$ > 0 were included to construct the DO-based disease similarity network, denoted as *G*^*D*^ *(V*^*D*^*, E*^*D*^). This process resulted in 806,505 weighted disease–disease similarity links (Fig 1(a)), which were represented in an adjacency matrix $A^{D}(m\times m)$, where each entry $A_{ij}^{D}=w_{ij}$ quantifies the similarity between diseases $d_{i}$ and $d_{j}$.

#### 2.2.2 A shared gene-based enhancer network.

We constructed the shared gene–based enhancer network (gEnhNet) based on known enhancer–target gene relationships from the DiseaseEnhancer database. In this framework, an enhancer is considered functionally associated with a disease if it targets one or more disease-associated genes. Functional similarity between two enhancers (*e*_*i*_ and *e*_*j*_) was quantified by the statistical significance of overlap between their target gene sets using the hypergeometric test:


$$\begin{array}{c} p=\sum {\mathrm{\backslash nolimits}}_{i=k}^{min(n_{j},n_{i})}\frac{\left(\begin{array}{c} n_{j}\\ k \end{array})(\begin{array}{c} n-n_{j}\\ n_{i}-k \end{array}\right)}{\left(\begin{array}{c} n\\ n_{i} \end{array}\right)} \end{array}$$

where *n* is the total number of genes annotated in DiseaseEnhancer, *n*_*i*_ and *n*_*j*_ denote the numbers of target genes for *e*_*i*_ and *e*_*j*_, and *k* is the number of shared target genes between them. This analysis directly considers overlapping genes between enhancer pairs without using any additional genomic window.

Enhancer pairs with *p ≤ 0.05* were considered significantly overlapping and retained as network edges. This process yielded 2,636 significant enhancer–enhancer links among 539 enhancers (Fig 1(b)), forming the adjacency matrix $A^{Eg}$ (*n*_*g*_ × *n*_*g*_), where each entry $A_{ij}^{Eg}=\text{1}$ if a functional interaction exists between enhancers $e_{i}$ and $e_{j}$, or 0 otherwise.

The DiseaseEnhancer database does not specify the methods (e.g., ABC, E2G, eQTL and CRISPRi) used to determine enhancer–gene links or provide tissue/cell-type–specific annotations. Therefore, this network captures general enhancer–gene associations across studies but does not account for cell-type–specific effects. Although EnhancerAtlas [11] provides such information, it lacks known disease–enhancer associations, making it unsuitable for this study.

#### 2.2.3 A heterogeneous network.

Following our previous approach (RWDisEnh) [19], we integrated the disease similarity network (Section 2.2.1 A disease similarity network) and the gEnhNet (Section 2.2.2 A shared gene-based enhancer network) through known disease–enhancer associations obtained from the DiseaseEnhancer database. In this framework, the disease and enhancer subnetworks are connected via a bipartite adjacency matrix $B^{Eg}$, where an element $B_{ij}^{Eg}=\text{1}$ indicates that enhancer e_i_ is known to be associated with disease d_j_, and 0 otherwise. The resulting heterogeneous network G^H^ (V^H^, E^H^) (Fig 1(d)), is represented by the adjacency matrix:


$$A^{H}=[\begin{array}{cc} A^{Eg} & B^{Eg}\\ {{(B}^{Eg})}^{T} & A^{D} \end{array}]$$

where $A^{Eg}$ and $A^{D}$ correspond to the enhancer–enhancer and disease–disease adjacency matrices, respectively.

This heterogeneous framework allows random walks to move not only within each subnetwork but also across them, thereby enabling the propagation of functional and semantic information between diseases and enhancers. Specifically, known disease–enhancer links serve as bridges through which network topology and biological context are jointly exploited to prioritize novel disease-associated enhancers.

#### 2.2.4 A sequence-based enhancer similarity network.

The collected enhancers, identified by their chromosomal positions, were first converted to BED format and then to FASTA sequences using BEDtools [25]. We then used Clustal Omega [24] to calculate pairwise similarity between enhancer sequences based on sequence alignment scores. After removing seven enhancers showing zero similarity with all others, a sequence-based enhancer similarity network (sEnhNet) consisting of 301,476 pairwise similarities among 777 enhancers was obtained (Fig 1(c)). The network is represented by an adjacency matrix $A^{Es}$ (*n*_*s*_ × *n*_*s*_), where each element $A_{ij}^{Es}$ denotes the sequence similarity score between enhancers *e*_*i*_ and *e*_*j*_. Since enhancer sequence annotations vary in completeness across studies, the quality of this network depends on the accuracy of the available enhancer sequence data. Nevertheless, incorporating this sequence-based layer complements the gEnhNet by capturing intrinsic sequence-level similarities among enhancers.

#### 2.2.5 A multiplex network.

In this study, we additionally built a multiplex enhancer network (MulNet) composed of two layers: gEnhNet ($A^{Eg}$ *(n*_*g*_× *n*_*g*_*)*) and sEnhNet ($A^{Es}$ *(n*_*s*_× *n*_*s*_*)*) (Fig 1(e)). Both layers share the same node set (*n*), representing the union of all enhancers across the two networks. Enhancers absent from one layer were added as isolated nodes to ensure structural consistency; thus, the two layers in the multiplex network are denoted as $A_{M}^{Eg}$ *(n* × *n)* and $A_{M}^{Es}$ *(n* × *n)*. The overall multiplex network is characterized by a block adjacency matrix $A^{M}(\text{2}n\times \text{2}n)$ [29,30]. This multiplex structure enables the integration of complementary biological information—functional similarity from shared target genes and sequence similarity from enhancer DNA sequences—within a unified multi-layer representation.

#### 2.2.6 A multiplex-heterogeneous network.

Finally, the MulNet was integrated with the disease similarity network (Section 2.2.1 A disease similarity network) via the known disease–enhancer associations, resulting in a multiplex–heterogeneous network (MulHetNet) of diseases *and* enhancers (Fig 1(f)). This comprehensive network allows information to propagate both across enhancer layers and between enhancers and diseases, supporting more robust identification of potential disease-associated enhancers.

### 2.3 Random walk with restart scheme on networks of diseases and enhancers

#### 2.3.1 A random walk with restart scheme.

Consider a weighted, connected network *G*(*V*, *E*), where *V*={*v*_*1*_*, v*_*2*_*, …, v*_*N*_} represents the nodes, *E*={(*v*_*i*_*, v*_*j*_)| *v*_*i*_*, v*_*j*_ ∈ *V*} denotes the edges, and *S* ⊆ *V* is the subset of source nodes. The network’s link weights are captured in an N × N adjacency matrix *A*. We describe a method to evaluate the significance of a node *v*_*i*_ relative to *S* using RWR, a modified random walk where a walker either transitions to a neighboring node or returns to the source nodes with a restart probability *γ*∈(0, 1). The RWR process is governed by:


$$P_{t+\text{1}}=(\text{1}-\gamma )MP_{t}+\gamma P_{\text{0}}$$

Here, $P_{t}$ is an N × 1 probability vector at time step *t*, with its *i*th entry indicating the likelihood of the walker being at node *v*_*i*_ ∈ *V*, and $P_{\text{0}}$ is the initial N × 1 probability vector. The transition matrix M, derived from column-normalizing *A*, has entries *(i, j)* reflecting the probability of moving from *v*_*i*_ to *v*_*j*_ among *v*_*i*_’s neighbors. Nodes are ultimately ranked based on the steady-state probability vector $P_{\infty }$, where each node’s steady-state value indicates its importance relative to *S*.

This ranking is applied to identify new associations between a disease of interest (*d*) and an enhancer (*e*), with enhancer rankings determined by their significance to *S*, reflecting their association strength with *d*. The RWR approach has previously been utilized for disease-gene association predictions [31–36].

#### 2.3.2 RWDisEnh.

As described previously [19], RWDisEnh employs a RWR approach to rank candidate enhancers. In gEnhNet, enhancers linked to a disease of interest define the source nodes, with probabilities updated iteratively. For the heterogeneous network, the RWR is extended to simultaneously rank enhancers and diseases, incorporating transition probabilities across networks. Full details are available in [19].

#### 2.3.3 RWDisEnh+.

In this study, the random walk with restart scheme was extended to work on the MulHetNet to predict novel disease-associated enhancers. The extension has previously been shown to be effective in predicting disease-associated genes [37].

First, we extend the random walk with restart scheme to the MulNet. The adjacency matrix $A^{M}$ of the multiplex network is defined as:


$$A^{M}=[\begin{array}{cc} (\text{1}-\delta )A_{M}^{Eg} & \delta I\\ \delta I & (\text{1}-\delta )A_{M}^{Es} \end{array}]$$

where I is the *n* × *n* identity matrix. The parameter δ ∈ [0,1] indicates the likelihood of remaining in a layer or transitioning between enhancer network layers.

We define the transition matrix $M^{M}$ as the column-normalized form of $A^{M}$. The RWR equation for the multiplex network is then expressed as:


$${\overset{―}{P}}_{t+\text{1}}=(\text{1}-\gamma )M^{M}{\overset{―}{P}}_{t}+\gamma {\overset{―}{P}}_{\text{0}}$$

where ${\overset{―}{P}}_{t+\text{1}}=[P_{t+\text{1}}^{Eg},P_{t+\text{1}}^{Es}]$ and ${\overset{―}{P}}_{t}=[P_{t}^{Eg},P_{t}^{Es}]$ are *n* × 2 vectors representing the probability distribution of the walker in the multiplex graph. ${\overset{―}{P}}_{\text{0}}$ represents the initial probability distribution and is defined as:


$${\overset{―}{P}}_{\text{0}}=[\tau P_{\text{0}}^{Eg},(\text{1}-\tau )P_{\text{0}}^{Es}]$$

where $P_{\text{0}}^{Eg}$ and $P_{\text{0}}^{Es}$ represent the initial probability of the shared gene-based enhancer network and sequence-based enhancer network, respectively. *τ* weighs the importance of each enhancer network.

For the MulHetNet, the matrix $A^{MH}$ is defined as:


$$A^{MH}=[\begin{array}{cc} A^{M} & B^{MH}\\ {{(B}^{MH})}^{T} & A^{D} \end{array}]$$

where $B^{MH}$ is the adjacency matrix of the network connecting diseases in the disease similarity network and enhancers in the two enhancer networks are identical, thus $B^{MH}$ can be defined as follows:


$$B^{MH}=(\begin{array}{c} B_{\left(n\times m\right)}^{Eg}\\ B_{\left(n\times m\right)}^{Es} \end{array})$$

where $B^{Eg}$ and $B^{Es}$ represent the adjacency matrices of the bipartite networks that link enhancers in gEnhNet and sEnhNet, respectively, to the disease similarity network via known disease-enhancer associations.

Then, the transition matrix $M^{MH}$ of MulHetNet can be calculated from $A^{MH}$ in the same way as for the heterogeneous network [36]. Thus, the RWR equation on the multiplex-heterogeneous network becomes:


$${\hat{P}}_{t+\text{1}}=(\text{1}-\gamma )M^{MH}{\hat{P}}_{t}+\gamma {\hat{P}}_{\text{0}}$$

where ${\hat{P}}_{t+\text{1}}$, ${\hat{P}}_{t}$ and ${\hat{P}}_{\text{0}}$ are now (2*n* + *m*) × 1 vectors since all enhancers in the MulNet and all diseases in the disease similarity network are ranked at the same time. Also, the initial probability vector becomes:


$${\hat{P}}_{\text{0}}=[\begin{array}{c} (\text{1}-\eta ){\overset{―}{P}}_{\text{0}}\\ \eta P_{\text{0}}^{D} \end{array}]$$

### 2.4 Performance evaluation

To evaluate the predictive performance of the ranking methods (RWDisEnh and RWDisEnh+) across various disease–enhancer networks, we applied a 3-fold cross-validation (CV) procedure independently for each disease. Consequently, only diseases linked to at least three enhancers were included in the validation, ensuring that each fold contained at least one enhancer. Specifically, for a given disease *d* with a set of known associated enhancers *S*, we randomly partitioned *S* into three approximately equal folds. In each iteration, one fold was reserved as the test set, while the remaining two served as source nodes (i.e., known associations) for training. If the number of enhancers for a disease was not divisible by three, folds contained slightly different sizes (e.g., 1–1–2 for four enhancers).

Predictive performance was assessed using the area under the receiver operating characteristic (ROC) curve (AUC), which quantifies the trade-off between sensitivity and specificity. A higher AUC value (closer to 1.0) indicates better discrimination of true disease–enhancer associations from non-associated pairs. We selected k = 3 to maximize the number of diseases eligible for evaluation, as higher k values (e.g., 5 or 10) would exclude many diseases with limited known enhancers.

### 2.5 Evidence support for novel predictions

To provide evidence supporting novel predictions (i.e., highly ranked enhancers not previously reported in DiseaseEnhancer) for each disease, we first selected the top *k*-ranked enhancers (with *k* ranging from 10 to 100) and searched for supporting evidence.

A disease–enhancer association was considered supported by evidence if one or more single nucleotide polymorphisms (SNPs) located within the enhancer region were significantly associated with the corresponding disease phenotype in genome-wide association studies (GWASs). For this purpose, we conducted a genomic region search using PhenoScanner V2 [38], a curated database of publicly available results from large-scale human genetic association studies. PhenoScanner V2 contains over 150 million genetic variants and more than 65 billion associations (compared to 350 million in PhenoScanner V1 [39]) involving diseases and traits, gene expression, metabolite and protein levels, and epigenetic markers. We retrieved SNP–phenotype associations using the PhenoScanner R package, querying all enhancer regions. To identify independent GWAS signals, linkage disequilibrium (LD) was calculated using the LDlinkR R package [40], and only the SNP with the lowest p-value was retained if its LD (r²) exceeded 0.2 with another SNP (as reflected in Table 2).

**Table 2:** GWAS-supported enhancer–disease associations identified among the top 10 ranked enhancers predicted by RWDisEnh+.

Disease	Enhancer	Target Genes	SNP ID (P-value)	Evidence Sources (PubMed ID/UK Biobank)
bronchial asthma	chr10:6074002–6104800	IL2RA	rs12722502 (1.903e-14), rs7910961 (2.728e-06), rs1924138 (5.471e-06)	UKBB
celiac disease	chr2:30439602–30453600	LBH	rs1355208 (2.035e-06)	21383967, 20190752
cardiovascular disease	chr9:21974127–21976127	CDKN2A	rs3731239 (1.916e-47), rs36228503 (2.624e-06), rs36228834 (1.833e-15), rs3731238 (2.997e-06)	26343387, 29212778, 28714975, 21378988
rheumatoid arthritis	chr14:69236402–69258800	ZFP36L1	rs12435329 (6.9e-08)	24390342
	chr17:37910411–37959400	IKZF3	rs10445308 (4.3e-12), rs112350333 (6.8e-06)	24390342
	chr4:26085002–26091000	RP11-263J14.1	rs10517086 (1.6e-16)	20453842, 24390342, 23143596
systemic lupus erythematosus	chr1:206941402–206947400	IL10	rs3024493 (6e-08)	26502338
type-2 diabetes	chr16:53799602–53801200	FTO	rs1421085 (4e-15)	26818947
ulcerative colitis	chr15:67360202–67451200	SMAD3	rs17293632 (2e-08)	28067908, 23128233, 26192919, 21297633
	chr17:37910411–37959400	IKZF3	rs12946510 (1e-25)	28067908, 23128233, 26192919
	chr17:40504602–40508200	STAT3	rs9891119 (2.721e-06)	26192919

*Note: only the SNP with the lowest p-value was retained if it had linkage disequilibrium (LD) > 0.2 with other SNPs*.

For enhancers that lacked direct GWAS evidence, we performed pathway enrichment analysis on genes whose genomic regions overlapped with the unevidenced enhancers. Specifically, we identified these genes using the GenomicRanges [41] and TxDb.Hsapiens.UCSC.hg38.knownGene R packages. Enrichment analysis was then carried out using the clusterProfiler [42] package, focusing on KEGG [43] pathways via the *enrichKEGG()* function. All KEGG-annotated human genes (9,447 genes, as reported by clusterProfiler) were used as the background, ensuring that enrichment results reflect specific biological processes associated with the predicted enhancers rather than general autoimmune or GWAS-driven patterns.

## 3. Results

### 3.1 Parameter settings

In our previous study, RWDisEnh demonstrated stable performance across variations in parameters controlling the random walk process. Here, we evaluated the effects of the restart probability (γ), the jumping probability between enhancer layers (δ), and the importance weight of enhancer layers (τ) on RWDisEnh+ . Each parameter was varied from 0.1 to 0.9 while the others were fixed at 0.5, and prediction performance was assessed using 3-fold cross-validation.

**Fig 2:**
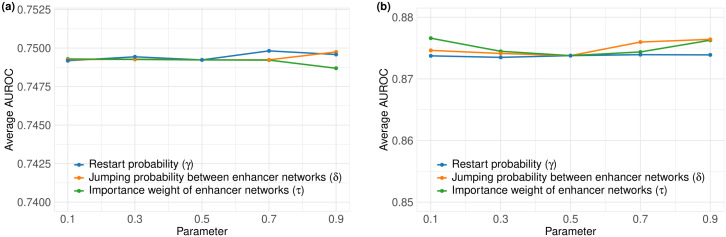
Predictive performance of RWDisEnh+ under various parameter settings. **(a)** Performance on the MulNet and **(b)** on the MulHetNet. Restart probability (γ) was varied in {0.1, 0.3, 0.5, 0.7, 0.9}, with other parameters fixed at 0.5. Jumping probability between enhancer layers (δ) was varied in {0.1, 0.3, 0.5, 0.7, 0.9}, with other parameters fixed at 0.5. Importance weight of enhancer layers (τ) was varied in {0.1, 0.3, 0.5, 0.7, 0.9}, with other parameters fixed at 0.5. Mean AUC values were calculated across all diseases for each parameter configuration.

As shown in Fig 2, RWDisEnh+ achieved consistent AUC values across all parameter settings—approximately 0.749 for the MulNet (Fig 2(a)) and 0.874–0.876 for the MulHetNet (Fig 2(b))—demonstrating strong robustness to parameter changes. Therefore, all parameters were fixed at 0.5 in subsequent analyses.

### 3.2 Sequence-based enhancer similarity network improves the prediction performance

In RWDisEnh, we demonstrated that the RWR scheme achieved the best prediction performance on the HetNet compared with running solely on the gEnhNet or the disease similarity network. It also outperformed two baseline methods: the network diffusion method PageRank with Priors (PRP) [21] and the neighborhood-based method MaxLink [22,23].

In this study, we evaluated the contribution of the sEnhNet to the overall prediction performance. Specifically, we compared the predictive performance of gEnhNet with that of the MulNet, in which sEnhNet was incorporated as an additional enhancer layer (Fig 1(e)). Similarly, we compared HetNet and the MulHetNet, where sEnhNet was added on top of the heterogeneous structure (Fig 1(f)). Prediction performance was assessed using the 3-fold cross-validation scheme, considering only diseases with at least three known associated enhancers. Due to the demonstrated stability of the RWR scheme across all networks, the parameters were fixed at γ = λ = δ = 0.5. The average AUC values across all diseases are summarized in Table 1. The results show that MulNet (AUC = 0.831) outperforms gEnhNet (AUC = 0.747), while MulHetNet (RWDisEnh+) (AUC = 0.874) surpasses HetNet (RWDisEnh) (AUC = 0.819) and achieves the best overall performance.

**Table 1:** Performance comparison between networks without and with the sequence-based enhancer similarity network.

Sequence-based enhancer similarity network (sEnhNet)	Networks	AUC values
Without	gEnhNet	0.747
	HetNet (**RWDisEnh**)	**0.819**
With	MulNet	0.831
	MulHetNet (**RWDisEnh+)**	**0.874**

These findings indicate that incorporating sequence-based enhancer similarity information (sEnhNet) enhances the ability of the model to identify disease–enhancer associations, providing complementary insights to those derived from gene-based enhancer relationships.

### 3.3 Prediction of novel disease-associated enhancers

We first evaluated the ability of RWDisEnh and RWDisEnh+ to predict novel disease–enhancer associations. Specifically, for each disease, we applied RWDisEnh on the HetNet and RWDisEnh+ on the MulHetNet to rank all candidate enhancers—that is, enhancers not previously known to be associated with the disease of interest. For each disease, we selected the top *k* ranked enhancers (with *k* ranging from 10 to 100) and searched for supporting evidence. A predicted disease–enhancer association was considered supported by evidence if one or more single nucleotide polymorphisms (SNPs) located within the enhancer region were significantly associated with the corresponding disease phenotype in genome-wide association studies (GWASs). To identify such associations, we conducted genomic region searches using PhenoScanner V2 (Section 2.5 Evidence support for novel predictions).

**Fig 3:**
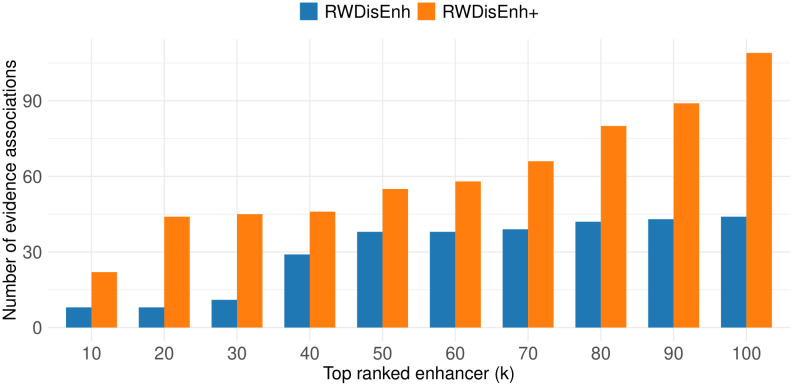
Comparison of the number of evidence-supported associations identified for top-ranked enhancers. The figure compares the number of GWAS-supported disease–enhancer associations retrieved by RWDisEnh+ and RWDisEnh across different top-*k* ranked enhancers.

The predictive capability of RWDisEnh (on the HetNet) and RWDisEnh+ (on the MulHetNet) was evaluated by counting the number of evidence-supported associations between the top-ranked enhancers and diseases identified through the PhenoScanner search. As shown in Fig 3, RWDisEnh+ consistently retrieved a higher number of GWAS-supported disease–enhancer associations than RWDisEnh across all *k* values, demonstrating the benefit of incorporating sequence-based enhancer information.

We further investigated the GWAS-supported disease–enhancer associations retrieved by RWDisEnh+ among the top 10 ranked enhancers (S1 Table). Table 2 summarizes 11 direct associations between seven diseases (asthma, celiac disease, cardiovascular disease, rheumatoid arthritis, systemic lupus erythematosus, type 2 diabetes, and ulcerative colitis) and 10 enhancers, after retaining only independent SNP signals (i.e., the SNP with the lowest *p*-value among those in linkage disequilibrium, LD > 0.2, with others).

These results highlight the biological relevance of the top-ranked enhancers and emphasize the role of regulatory elements in complex diseases. Autoimmune diseases are particularly prominent in this dataset. For example, in rheumatoid arthritis, multiple enhancers show strong associations. Enhancers linked to ZFP36L1, IKZF3, and RP11-263J14.1 genes demonstrate robust connections to the disease, supported by several independent SNPs [44] (PubMed ID: 24390342). Interestingly, the SMAD3-associated enhancer (chr15:67360202–67451200), IKZF3-associated enhancer (chr17:37910411–37959400), and STAT3-associated enhancer (chr17:40504602–40508200) also exhibit significant associations with ulcerative colitis [45–47] (PubMed IDs: 28067908, 23128233, 26192919). This observation is consistent with previous reports of extensive genetic correlation among autoimmune disorders, in which multiple diseases share common susceptibility loci [48–50]. For cardiovascular disease, a strong association was observed with an enhancer linked to the CDKN2A gene, supported by multiple SNPs and several independent studies [51–54] (PubMed IDs: 26343387, 29212778, 28714975, 21378988), underscoring the importance of this regulatory region in cardiovascular health. For metabolic disorders, an association was identified between type 2 diabetes and an enhancer located within the FTO gene [55] (PubMed ID: 26818947). Although these signals reside in FTO introns, multiple studies have shown that they functionally regulate IRX3/IRX5 rather than FTO itself. Our model identifies enhancer–disease links independent of downstream target-gene interpretation, consistent with previous GWAS findings at this locus. In celiac disease, the predicted enhancer near the LBH gene [56,57] (PubMed IDs: 21383967, 20190752) shows strong GWAS support, while systemic lupus erythematosus was linked to an enhancer in the IL10 region [58] (PubMed ID: 26502338), both consistent with established autoimmune mechanisms. Finally, for bronchial asthma, an enhancer associated with the IL2RA gene showed multiple SNP associations (UK Biobank data), demonstrating the utility of large-scale population resources for validating predicted disease–enhancer links.

In summary, these results confirm that RWDisEnh+ successfully prioritizes biologically meaningful enhancer–disease associations supported by GWAS data across diverse disease categories. The multiple independent SNP associations provide a strong rationale for future functional studies to elucidate the mechanisms by which these enhancers influence disease susceptibility and to explore their potential as therapeutic targets.

For the top 10 ranked enhancers that lacked GWAS support, we conducted pathway enrichment analysis on the genes whose genomic regions overlapped these enhancers (Section 2.5 Evidence support for novel predictions). This analysis identified 23 diseases with at least one significantly enriched pathway (S2 Table). Table 3 summarizes representative results for three diseases listed in Table 1—bronchial asthma, celiac disease, and ulcerative colitis—illustrating potential biological relevance of the predicted enhancers.

**Table 3:** Pathway enrichment analysis for GWAS-unreported enhancers in three representative diseases.

Disease	Enhancer	Gene	Pathway (KEGG ID)
bronchial asthma	chr2:219965238–219974238, chr1:206941402–206947400, chr6:12902202–12905800, chr2:60691402–60718400, chrX:37639002–37678400, chr4:145487334–145489334, chrX:70441002–70448800, chr17:64299802–64307800, chr2:175626602–175632200	LOC105373891, PHACTR1, LANCL3, TSPAN7, SMAD1, DLG3, LOC124907909	Hippo signaling pathway (hsa04390)
			Transcriptional misregulation in cancer (hsa05202)
celiac disease	chr15:67360202–67451200, chr4:26085002–26091000, chr17:37910411–37959400, chr14:75986202–76011400, chr12:6420402–6449200, chr14:69236402–69258800, chr17:40504602–40508200, chr16:11440602–11455000, chr1:117095802–117109400	IFT43, CD27-AS1, CD27, LOC400499, TTF2	Thyroid hormone synthesis (hsa04918)
ulcerative colitis	chr2:30439602–30453600, chr4:26085002–26091000, chr14:75986202–76011400, chr12:6420402–6449200, chr14:69236402–69258800, chr16:11440602–11455000, chr1:117095802–117109400	IFT43, CD27-AS1, CD27, LOC400499, TTF2	Glycerolipid metabolism (hsa00561)
			Thyroid hormone synthesis (hsa04918)
			Glycerophospholipid metabolism (hsa00564)

For bronchial asthma, several enhancers overlapped genes such as SMAD1, TSPAN7, DLG3, and PHACTR1, which were significantly enriched in the Hippo signaling pathway (hsa04390) and Transcriptional misregulation in cancer (hsa05202). The Hippo pathway has been implicated in epithelial regeneration and tissue remodeling in the airway, processes that are dysregulated in chronic asthma.

Moreover, genes involved in transcriptional regulation and cell proliferation, such as SMAD1 (a downstream effector of BMP signaling), may contribute to the airway remodeling and immune regulation characteristic of asthma pathogenesis. These findings suggest that the predicted enhancers could influence asthma risk through modulation of epithelial cell growth and inflammatory signaling pathways.

In celiac disease, the genes CD27 and TTF2, located near the predicted enhancers, were enriched in the Thyroid hormone synthesis pathway (hsa04918). Although this pathway is not directly immune-specific, thyroid hormone dysregulation and autoimmune thyroiditis are commonly comorbid with celiac disease, indicating shared autoimmune mechanisms and genetic susceptibility. The presence of CD27, a co-stimulatory molecule expressed on T and B cells, further supports the relevance of immune signaling and lymphocyte activation in celiac pathogenesis.

For ulcerative colitis, genes including CD27, TTF2, and IFT43 were enriched in Glycerolipid metabolism (hsa00561), Glycerophospholipid metabolism (hsa00564), and again Thyroid hormone synthesis (hsa04918). Alterations in lipid metabolism have been linked to inflammatory bowel diseases, influencing membrane signaling, cytokine release, and epithelial barrier integrity.

In particular, glycerophospholipids serve as precursors for pro-inflammatory mediators, suggesting that the identified enhancers may regulate genes involved in lipid-driven inflammatory processes relevant to ulcerative colitis.

Collectively, these pathway enrichment results provide functional context for the novel enhancer–disease associations predicted by RWDisEnh+ . Even in the absence of direct GWAS support, the implicated genes and pathways align with biological mechanisms previously reported for these diseases, reinforcing the potential relevance of the newly predicted enhancer regions.

## 4. Conclusions and discussion

In this study, we developed RWDisEnh+ , an improved random walk with restart–based approach for predicting disease-associated enhancers by integrating heterogeneous and multiplex network structures. Specifically, we constructed a multiplex-heterogeneous network combining disease similarity, enhancer functional relationships based on shared target genes, and enhancer sequence similarity. The inclusion of the sequence-based enhancer similarity network significantly improved predictive performance compared with previous frameworks, as demonstrated by higher AUC values in cross-validation experiments. Moreover, the proposed model successfully identified novel disease-enhancer associations, many of which were supported by genome-wide association study (GWAS) evidence or functional pathway enrichment analysis.

Our results provide evidence that incorporating enhancer sequence similarity strengthens disease-enhancer association prediction, suggesting that enhancer-level sequence features encode functional relevance beyond gene-level regulatory networks. The identified associations shed light on disease mechanisms across immune, metabolic, and cardiovascular disorders, supporting the hypothesis that regulatory variants play key roles in disease pathogenesis. Notably, several autoimmune diseases (e.g., rheumatoid arthritis, ulcerative colitis, and systemic lupus erythematosus) share enhancer loci targeting key immune-regulatory genes such as IKZF3, SMAD3, and STAT3, which is consistent with previously established findings that autoimmune conditions exhibit extensive genetic correlations and overlapping pathogenic pathways.

Despite these promising findings, several limitations should be acknowledged. First, the approach relies on enhancer–gene relationships provided by the DiseaseEnhancer database, which primarily reflects genomic proximity or curated evidence. As a result, long-range enhancer–target interactions—spanning hundreds of kilobases or even megabases and detectable only through chromatin conformation or functional perturbation assays—may not be captured. This limitation means that some predicted enhancer–disease associations may involve regulatory mechanisms not explicitly represented in the enhancer–gene mapping used here. Future versions of RWDisEnh+ could integrate high-resolution chromatin interaction datasets (e.g., Hi-C [59], promoter capture Hi-C, ChIA-PET [60]) or functional enhancer–gene mapping frameworks (e.g., ABC, E2G, CRISPR-based screens) to more accurately model distal regulatory relationships. Second, the accuracy of the sequence-based enhancer similarity network depends on the quality and completeness of enhancer sequence annotations. Incomplete or low-quality annotations could affect the precision of similarity measures and downstream predictions. Third, our model does not yet account for the dynamic and tissue- or cell-type–specific activity of enhancers, which can vary across developmental stages and biological contexts. Integrating context-specific enhancer activity profiles from resources such as EnhancerAtlas [11] could improve the specificity and biological interpretability of predictions.

In the future, expanding RWDisEnh+ to integrate multi-omics data (e.g., chromatin accessibility, histone modifications, and eQTL information) and dynamic enhancer activity could further enhance its power to identify functionally relevant regulatory elements and uncover causal mechanisms underlying complex diseases. In addition, recent multiplex network–based propagation methods such as NetWalkRank [61]—although originally developed for cancer driver gene prioritization—could be adapted to the enhancer–disease prediction problem and incorporated into future extensions of our framework. Overall, RWDisEnh+ provides a flexible network-based framework that can be readily extended as more comprehensive regulatory datasets become available.

## Supporting information

S1 Table Detailed GWAS evidence for SNPs located within the top 10 enhancers predicted by RWDisEnh+. (XLSX)

S2 Table KEGG pathway enrichment analysis for genes overlapping top 10 ranked enhancers without GWAS support. (TXT)
